# The Function and Significance of SELENBP1 Downregulation in Human Bronchial Epithelial Carcinogenic Process

**DOI:** 10.1371/journal.pone.0071865

**Published:** 2013-08-19

**Authors:** Gu-Qing Zeng, Hong Yi, Peng-Fei Zhang, Xin-Hui Li, Rong Hu, Mao-Yu Li, Cui Li, Jia-Quan Qu, Xingming Deng, Zhi-Qiang Xiao

**Affiliations:** 1 Key Laboratory of Cancer Proteomics of Chinese Ministry of Health, Xiangya Hospital, Central South University, Changsha, Hunan, China; 2 School of Nursing, University of South China, Hengyang, Hunan, China; 3 Departments of Radiation Oncology, Emory University School of Medicine and Winship Cancer Institute of Emory University, Atlanta, Georgia, United States of America; South China Sun Yat-sen University Cancer Center, China

## Abstract

**Background:**

Our quantitative proteomic study showed that selenium-binding protein 1 (SELENBP1) was progressively decreased in human bronchial epithelial carcinogenic process. However, there is little information on expression and function of SELENBP1 during human lung squamous cell cancer (LSCC) carcinogenesis.

**Methods:**

iTRAQ-tagging combined with 2D LC-MS/MS analysis was used to identify differentially expressed proteins in the human bronchial epithelial carcinogenic process. SELENBP1, member of selenoproteins family and progressively downregulated in this process, was selected to further study. Both Western blotting and immunohistochemistry were performed to detect SELENBP1 expression in independent sets of tissues of bronchial epithelial carcinogenesis, and ability of SELENBP1 for discriminating NBE (normal bronchial epithelium) from preneoplastic lesions from invasive LSCC was evaluated. The effects of SELENBP1 downregulation on the susceptibility of benzo(a)pyrene (B[a]P)-induced human bronchial epithelial cell transformation were determined.

**Results:**

102 differentially expressed proteins were identified by quantitative proteomics, and SELENBP1 was found and confirmed being progressively decreased in the human bronchial epithelial carcinogenic process. The sensitivity and specificity of SELENBP1 were 80% and 79% in discriminating NBE from preneoplastic lesions, 79% and 82% in discriminating NBE from invasive LSCC, and 77% and 71% in discriminating preneoplastic lesions from invasive LSCC, respectively. Furthermore, knockdown of SELENBP1 in immortalized human bronchial epithelial cell line 16HBE cells significantly increased the efficiency of B[a]P-induced cell transformation.

**Conclusions:**

The present data shows for the first time that decreased SELENBP1 is an early event in LSCC, increases B[a]P-induced human bronchial epithelial cell transformation, and might serve as a novel potential biomarker for early detection of LSCC.

## Introduction

Lung cancer is the most frequently occurring malignancy with increasing incidence and is the leading cause of mortality in cancer-related deaths in China and worldwide [1], [2]. Although great improvement has been made in diagnosis and treatment of lung cancer, the overall patients' survival is still very low and does not exceed 15% [3]. The poor prognosis of this cancer is mainly explained by the fact that the limited understanding of its carcinogenic mechanisms and the diagnosis is generally made only at advanced stages.

Lung squamous cell carcinoma (LSCC) originates from the bronchial epithelial cells and is the most common histological type of lung cancer. It is known that carcinogenesis of LSCC is a multistage process [4]. With exposure to environmental carcinogens, bronchial epithelial carcinogenesis often progresses in the following manner: hyperplasia, squamous metaplasia (SM), atypical hyperplasia (AH), cancer *in situ* (CIS) and invasive cancer [5]. Currently, the mechanism of carcinogenesis of bronchial epithelial cells is still unclear, and there are no clinically established biomarkers available for early detection of LSCC.

LSCC is the end-point of a whole range of morphological abnormalities that are displayed in the bronchial epithelia of the patients with LSCC and/or smokers [5], and that could be used to identify key proteins associated with the ongoing carcinogenic process. Recently, we performed iTRAQ (isobaric tags for relative and absolute quantitation)-tagging combined with 2D LC-MS/MS (two-dimensional liquid chromatography-mass spectrometry) analysis to identify differentially expressed proteins in human bronchial epithelial carcinogenic process using laser capture microdissection-purified NBE (normal bronchial epithelium), SM, AH, CIS and invasive LSCC [6]. MS analysis found that selenium-binding protein 1 (SELENBP1) expression was progressively decreased in human bronchial epithelial carcinogenic process.

SELENBP1, a member of selenoproteins family, has been shown to bind selenium covalently [7], [8], and mediate the intracellular transport of selenium [9]. Epidemiological and clinical trial showed that a deficiency of dietary selenium is associated with an increased incidence of epithelial cancers including lung cancer [10], [11]. Selenium exerts its anticarcinogenic effects mainly through selenoproteins at nutritional levels. Therefore, SELENBP1 downregulation may play a critical role in regulating malignant transformation and cancer progression. However, there is little information on expression and function of SELENBP1 during human LSCC carcinogenic process.

Polycyclic aromatic hydrocarbons such as benzo(a)pyrene (B[a]P) are main lung carcinogens within tobacco smoke [12], and the source of DNA adducts [13]. It has been reported that selenium can inhibit carcinogen-induced covalent DNA adduct formation [14]–[17], promote DNA repair [18]–[20], and activate early barriers of tumorigenesis [21], indicating selenium can antagonize B[a]P-induced tumorigenesis. Because the anticarcinogenic effects of selenium are mediated possibly by SELENBP1, SELENBP1 downregulation is involved in LSCC carcinogenesis via increasing the susceptibility of human bronchial epithelial cells to B[a]P-induced tumorigenesis.

To reveal the function and significance of SELENBP1 during human LSCC carcinogenic process, the expressional changes of SELENBP1 during human bronchial epithelial carcinogenesis were detected, the value of SELENBP1 for early detection of LSCC were assessed, and the effect of SELENBP1 knockdown on the susceptibility of B[a]P-induced cell transformation in the immortalized human bronchial epithelial cell line 16HBE were analyzed.

Our results first time show that decreased SELENBP1 is an early event in LSCC carcinogenesis, and increases B[a]P-induced human bronchial epithelial cell transformation, and the expressional levels of SELENBP1 proteins could discriminate normal bronchial epithelium from preneoplastic lesions from invasive LSCC. Our data suggest that the detection of SELENBP1 expression can be used for monitoring high risk group of LSCC including smokers, and improving the early diagnosis of LSCC.

## Materials and Methods

### Sample Collection, Laser Capture Microdissection and Protein Extraction

All tissues from the LSCC patients undergoing curative surgery and receiving neither chemotherapy nor radiotherapy were obtained from Department of Cardiothoracic Surgery, Xiangya Hospitals of Central South University, China. The patients signed an informed consent form for the study which was approved by the ethical committee of Xiangya Hospital, Central South University. After surgery, tumor tissues and bronchi were removed from the resected pulmonary lobes, and stored at −80°C. Normal bronchial epithelium (NBE), squamous metaplasia (SM), atypical hyperplasia (AH), carcinoma in situ (CIS) and invasive LSCC were obtained from the bronchi or tumor tissues, and diagnosed by pathological examination of a H.E.-stained frozen tissue sections according to the 1999 World Health Organization/International Association for the Study of Lung Cancer classification [22]. LCM was performed with a Leica AS LMD system to purify the interest cells from each type of tissues as previously described by us [23].

Proteins were extracted from the microdissected cells as previously described by us [6]. To diminish the effect of sample biological variation on the results of a proteomics analysis, equal amounts of proteins from the microdissected cells of ten different individuals were pooled to generate one common sample for each type of tissues(NBE, SM, AH/CIS, and invasive LSCC), therefore we got the four pooled protein samples used for iTRAQ labeling.

### Protein Digestion and Labeling with iTRAQ Reagents

Trypsin digestion and iTRAQ labeling of proteins were performed according to the manufacturer’s protocol (Applied Biosystems). Briefly, 100 µg protein of each pooled sample was reduced and alkylated, and then digested overnight at 37°C with trypsin and labeled with iTRAQ™ reagents (Applied Biosystems) as follows: NBE, iTRAQ reagent 117; SM, iTRAQ reagents 114; AH/CIS, iTRAQ reagents 116; and invasive LSCC, iTRAQ reagent 115. Four labeled digests were then mixed.

### Off-line 2D LC-MS/MS

The mixed peptides were separated by strong cation exchange (SCX) and reverse-phase (RB) chromatography on a 20AD HPLC system (Shimadzu), and analyzed on Qstar XL (Applied Biosystems) as previously described by us [6]. The iTRAQ labeled peptides fragmented under CID conditions to give reporter ions at 114.1, 115.1, 116.1, and 117.1 Th. The ratios of peak areas of the iTRAQ reporter ions reflect the relative abundances of the peptides and, consequently, the proteins in the samples. Larger, sequence-information-rich fragment ions were also produced under these MS/MS conditions and gave the identity of the protein from which the peptide originated. iTRAQ labeling followed by 2D LC-MS/MS analysis was repeated in triplicate to diminish the effect of experimental variation on the results of a proteomics analysis.

### Proteomic Data Analysis

Protein identification and quantitation have been described by us [6]. Briefly, the software used for data acquisition was Analyst QS 1.1 (Applied Biosystems). The software used for protein identification and quantitation was ProteinPilot ™ 3.0 software (Applied Biosystems) with the integrated Paragon™ search algorithm and Pro Group™ algorithm (Applied Biosystems). Identified proteins were grouped by the software to minimize redundancy. All peptides used for the calculation of protein ratios were unique to the given protein or proteins within the group, and peptides that were common to other isoforms or proteins of the same family were ignored. The protein confidence threshold cutoff is 1.3 (unused ProtScore) with at least one peptide with 95% confidence. The average iTRAQ ratios from the triplicate experiments were calculated for each protein.

### Western Blotting

Western blotting was performed in one independent set of microdissected tissues (NBE, SM, AH, CIS and invasive LSCC, each 10 cases). Briefly, 30 µg of lysates were separated by 10% SDS-PAGE, and transferred to PVDF membranes. Blots were incubated with primary anti-SELENBP1 antibody (1∶500; Sigma) overnight at 4°C, followed by incubation with a horseradish peroxidase-conjugated secondary antibody (1∶3000; Amersham Biosciences) for 1 h at room temperature. The signal was visualized with ECL detection reagent (Amersham Biosciences) and quantitated by densitometry using ImageQuant image analysis system (Storm Optical Scanner, Molecular Dynamics). β-actin was simultaneously detected using mouse anti-β-actin antibody (1∶3000; Sigma) as a loading control.

### Immunohistochemistry and Evaluation of Staining

An independent set of formalin-fixed and paraffin-embedded archival tissue specimens including 66 cases of NBE, 64 cases of SM, 60 cases of AH, 13 cases of CIS, 66 cases of invasive LSCC was obtained from bronchoscopic or surgical procedures at the People Hospital of Hunan Province, Changsha, China., and used for SELENBP1 immunohistochemical staining. The patients recruited in this study received neither chemotherapy nor radiotherapy. The parameters of patients and tissue specimens are shown in Table S1. Immunohistochemistry were done on formalin-fixed and paraffin-embedded tissue sections using a standard immunohistochemical technique.

Immunostaining was blindly evaluated by two investigators in an effort to provide a consensus on staining patterns by light microscopy. A quantitative score was performed by adding the score of staining area and the score of staining intensity for each case to assess the expression levels of SELENBP1 protein as previously described by us [24]. A combined staining score of ≤2 was considered to be low staining (no expression); a score between 3 and 4 was considered to be moderate staining (expression); that between 5 and 6 was considered to be strong staining (high expression).

### Statistical Aanalysis of Immunohistochemical Data

All statistical analyses were performed using SPSS 15.0 software. Difference of SELENBP1 expression between the two stages of bronchial epithelial carcinogenesis (SM *vs.* NBE; AH/CIS *vs.* SM; LSCC *vs.* AH/CIS; LSCC *vs.* NBE) was analyzed using Mann-Whitney U test. Due to the small number of CIS, it was combined with AH into one group. Moreover, SELENBP1 protein was assessed for its ability to discriminate NBE from preneoplastic lesions (SM, AH and CIS) from invasive LSCC by evaluating its ROC curve based on the immunohistochemistry scores as previously described by us [6], [24]. Sensitivity, specificity, positive predictive value, and negative predictive value of SELENBP1 protein were calculated. A two-sided P<0.05 was considered significant.

### Stable Transfection of SELENBP1 siRNA Plasmid into Human Bronchial Epithelial Cells

Three SELENBP1 siRNA plasmids pLKO.1-SELENBP1-shRNAs, each of which includes one specific shRNA for SELENBP1, and empty vector pLKO.1 were purchased from Openbiosystems. For stable transfection, 1×10^7^ immortalized human bronchial epithelial cell line 16HBE cells were transfected with pLKO.1-SELENBP1-shRNA and pLKO.1 using Lipofectamine 2000 Reagent (Invitrogen), respectively, according to the manufacturer’s instructions. After 14 days of selection in DMEM medium containing 1.0 µg/ml puromycin (Invitrogen), individual puromycin-resistant colonies were isolated and expanded. The expression of SELENBP1 in these clones was determined by Western blotting.

### Cell Culture and Carcinogen Exposures

Stably transfected 16HBE cells with pLKO.1-SELENBP1-shRNAs and empty vector, and untransfected cells were cultured to 30–40% confluence in DMEM medium (Invitrogen) supplemented with 10% FBS (Invitrogen). The cells were exposed to 1 µm B[a]P(Sigma) or vehicle (DMSO; Sigma) for 1 day and then recovered in fresh medium without B[a]P for 6 days. After repeated treatment with B[a]P or DMSO for 18 weeks, the cells were harvested, and subjected to analyses of cell transformation characteristics including cell proliferation, anchorage dependent and independent colony formation, cell cycle and apoptosis.

### Analysis of Cell Growth in Low Serum Medium by MTT Assay

The cells in DMEM medium containing 0.5% FCS were plated at 1×10^4^ cells per well in 96-well tissue culture plates, and grew for 7 days. Every 24 h, 20 µl of MTT (5 mg/ml; Sigma) was added to wells, and the medium was removed after 4 h of incubation. 150 µl DMSO was added to each well for 10 min at room temperature. The absorbance of each well was read with a Bio-Tek Instruments EL310 Microplate Autoreader at 490 nm. MTT assay was performed three times in triplicate.

### Anchorage Dependent and Independent Colony Formation Assays

Plate and soft agar colony formation assays were performed to detect anchorage dependent and independen cell growth, respectively, as previously described by us [6]. All assays were performed three times in triplicate.

### Flow Cytometry Analysis

Analyses of cell cycle and apoptosis by flow cytometry were performed as previously described by us [6]. For cell cycle analysis, the cell grew in DMEM medium containing 10% FCS for 24 h, and for cell apoptosis analysis, the cells grew in serum free DMEM medium for 24 h. Three independent experiments were done.

### Hoechst 33258 Staining of Apoptotic Cells

The cells were cultured with DMEM medium containing 10% FCS in 6-well tissue culture plates for 24 h and then cultured in serum free DMEM medium for another 24 h, washed with PBS, fixed with 4% paraformaldehyde for 30 min at 4°C, and stained with 5 µg/ml cell-permeable DNA dye Hoechst 33258 (Sigma) dissolved in Hanks’ buffer in the dark for 10 min. Apoptotic cells were identified on the basis of the presence of highly condensed or fragmented nuclei. To calculate the percentage of apoptotic cells, at least 200 cells from three randomized microscopic fields were counted.

## Results

### Progressive Downregulation of SELENBP1 Expression during Human Bronchial Epithelial Carcinogenesis

**Figure 1:**
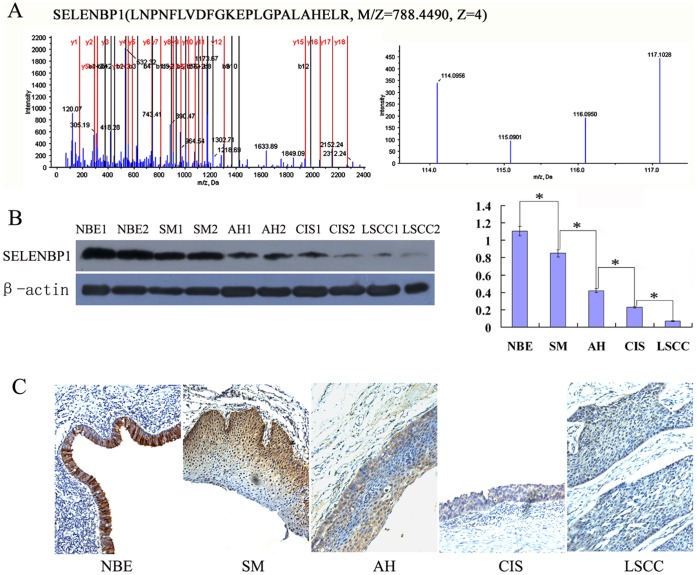
Expressional changes of SELENBP1 in the human bronchial epithelial carcinogenic process. **A**, MS/MS spectra used for the identification and quantitation of SELENBP1. (*left*) the sequence LNPNFLVDFGKEPLGPALAHELR allows the identification of SELENBP1. (*right*) the released iTRAQ reporter ions provide the relative quantitation of SELENBP1 from the four tissues evaluated. NBE, labeled with iTRAQ reagent 117; SM, labeled with iTRAQ reagents 114, AH/CIS, labeled with iTRAQ reagent 116; and invasive LSCC, labeled with iTRAQ reagent 115. **B**, detection of SELENBP1 expression in the microdissected tissues by Western blotting. (*left*) a representative result of Western blotting shows the expressions of SELENBP1 in the microdissected NBE, SM, AH, CIS and invasive LSCC; (*rig*ht) histogram shows the expression levels of SELENBP1 in these tissues as determined by densitometric analysis. β-actin is used as the internal loading control. Columns, mean from 10 cases of tissues; bars, S.D. (*, *P*<0.05 by One-way ANOVA). **C**, detection of SELENBP1 expression in the formalin-fixed and paraffin-embedded archival tissue specimens by immunohistochemistry. A representative result of immunohistochemistry shows the expression of SELENBP1 in the NBE, SM, AH, CIS and invasive LSCC. Original magnification, ×200.

The iTRAQ Labeling and 2D LC-MS/MS analysis resulted in identification of 102 differentially expressed proteins in at least one of the two-stage comparisons (NBE vs. SM, AH/CIS or LSCC; SM vs. AH/CIS or LSCC; AH/CIS vs. LSCC) except differentially expressed in NBE and LSCC. The names and detailed information of these 102 proteins were shown in Table S2. Among these differential proteins, SELENBP1 was progressively downregulated during the human bronchial epithelial carcinogenic process (Figure 1A). To validate the proteomic results, Western blotting was performed to detect the expressional levels of SELENBP1 in an independent set of LCM-purified tissue samples including NBE, SM, AH, CIS and invasive LSCC, and immunohistochemistry was performed to detect the expressional levels of SELENBP1 in an independent set of archival tissue specimens including NBE, SM, AH, CIS, and invasive LSCC. The results of both Western blotting and immunohistochemistry showed that SELENBP1 expression was progressively decreased along with evolution of bronchial epithelial carcinogenesis (Figure 1B and C, Table 1), which is consistent with the findings in proteomic analysis. Moreover, there was significantly different in the expressional levels of SELENBP1 in the two successive stages of bronchial epithelial carcinogenic process (Table 1).

**Table 1:** The difference of SELENBP1 expression in bronchial epithelial carcinogenic process.

	n	Score		
		Low(0–2)	Moderate(3–4)	High(5–6)
SELENBP1				
NBE	66	9	24	33
SM	64	16	27	21
AH/CIS	73	30	29	14
LSCC	66	42	18	6

The *P*-value of NBE vs. SM, SM vs. AH/CIS, AH/CIS vs. LSSC and NBE vs. LSSC was 0.030, 0.024, 0.007, and 0.000, respectively, by Mann-Whitney U test.

### Ability of SELENBP1 as A Biomarker for Discriminating NBE from Preneoplastic Lesions from Invasive LSCC

**Figure 2:**
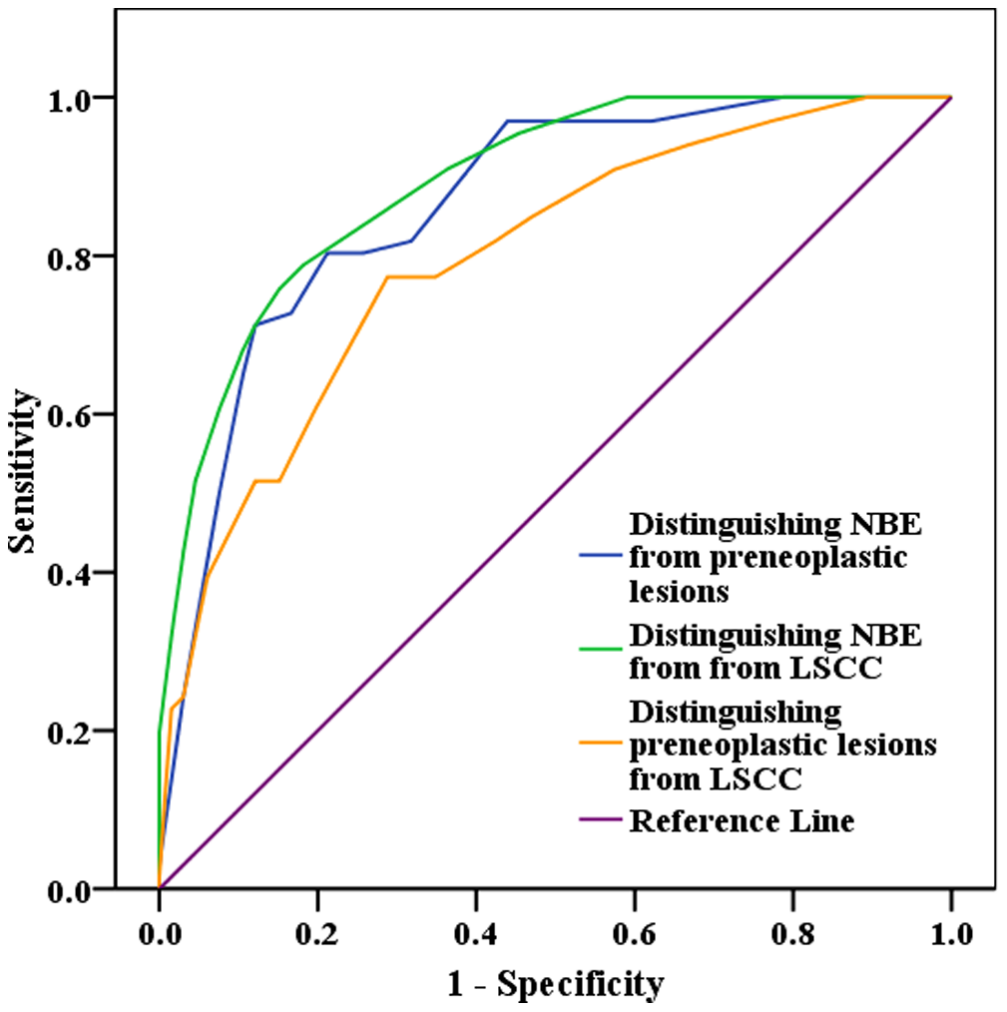
Efficacy of SELENBP1 in discriminating NBE from preneoplastic lesions(SM, AH and CIS) from invasive LSCC. Receiver operating characteristic (ROC) curves of SELENBP1 in discriminating NBE from preneoplastic lesions, NBE from invasive LSCC, and preneoplastic lesions from invasive LSCC.

The ability of SELENBP1 protein levels in distinguishing NBE from preneoplastic lesions (SM, AH and CIS) from invasive LSCC was analyzed by determining its ROC curves. The area under the curve (AUC) of SELENBP1 protein is listed in Table 2 together with its individual values of merit. When SELENBP1 protein serves as a biomarker, its sensitivity and specificity are 80% and 79% in discriminating NBE from preneoplastic lesions, 79% and 82% in discriminating NBE from invasive LSCC, and 77% and 71% in discriminating preneoplastic lesions from invasive LSCC, respectively (Figure 2, Table 2). The results strongly support that progressive decrease of SELENBP1 is involved in human bronchial epithelial carcinogenic process, and suggest that SELENBP1 downregulation is an early event in LSCC carcinogenesis, and might serve as a novel potential biomarker for early detection of LSCC.

**Table 2:** Receiver operating characteristics from IHC scores of SELENBP1 in distinguishing NBE from preneoplastic lesions from invasive LSCC.

	Sensitivity	Specificity	PPV	NPV	AUC
Distinguishing NBE from preneoplastic lesions	0.80	0.79	0.69	0.84	0.86
Distinguishing NBE from LSCC	0.79	0.82	0.82	0.79	0.89
Distinguishing preneoplastic lesions from LSCC	0.77	0.71	0.65	0.88	0.80

PPV: positive predict value; NPV: negative predict value; AUC: area under the curve.

### Knockdown of SELENBP1 Increased the Susceptibility of B[a]P-induced Human Bronchial Epithelial Cell Transformation

**Figure 3:**
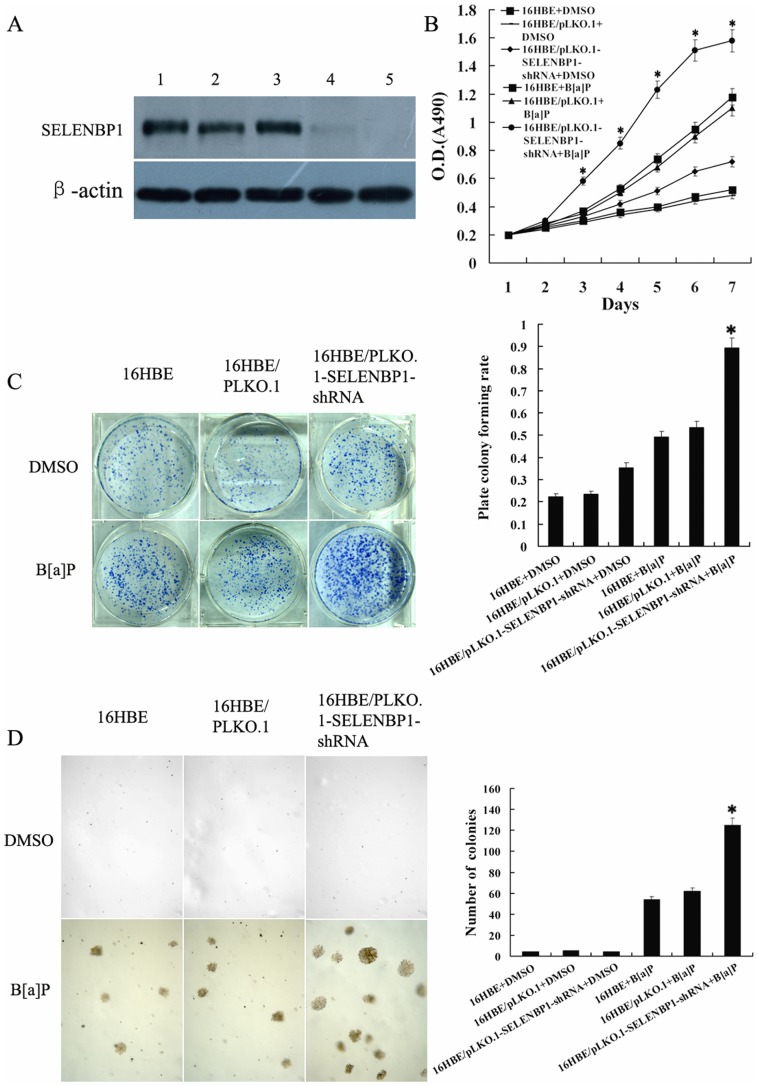
The effects of SELENBP1 gene knockout on the B[a]P-induced human bronchial epithelial cell transformation. **A**, Western blotting shows SELENBP1 expression in the untransfected (1), empty vector pLKO.1-transfected (2, 3) and pLKO.1-SELENBP1-shRNA-tansfected 16HBE cells (4, 5). β-actin is used as an internal control for loading. **B**, cell growth in low serum medium after exposed to B[a]P for 18 weeks. Cells were subjected to MTT assay as described in “Materials and Methods”. Three experiments were done; points, mean; bars, S.D. (*, *P*<0.05 vs. untransfected or empty vector -transfected 16HBE cells after exposed to B[a]P by Student’s *t* test). C, anchorage dependent colony growth after cells exposed to B[a]P for 18 weeks. (*left*) cells were subjected to plate colony formation assay as described in “Materials and Methods”, and colonies were stained with crystal violet and photographed under camera; (*right*) the histogram showed plate colony formation rates. Three experiments were done; columns, mean; bars, S.D. (**, *P*<0.05 *vs.* untransfected or empty vector -transfected 16HBE cells after exposed to B[a]P by One-way ANOVA). D, anchorage independent colony growth after cells exposed to B[a]P for 18 weeks. (*left*) cells were subjected to soft agar colony formation assay as described in “Materials and Methods”, and colonies were photographed under microscope; (*right*) the histogram showed number of soft agar colonies in 10 randomly chosen microscopic fields using a 5× objective. Three experiments were done; columns, mean; bars, S.D. (*, *P*<0.05 *vs.* untransfected or empty vector -transfected 16HBE cells after exposed to B[a]P by One-way ANOVA). Cell proliferation, and plate and soft agar colony growth of the cells exposed to vehicle (DMSO) for 18 weeks are also shown and used as controls. 16HBE, untransfected cells; 16HBE/pLKO.1, empty vector pLKO.1-transfected cells; 16HBE/pLKO.1-SELENBP1-shRNA, pLKO.1-SELENBP1-shRNA -tansfected cells.

**Figure 4:**
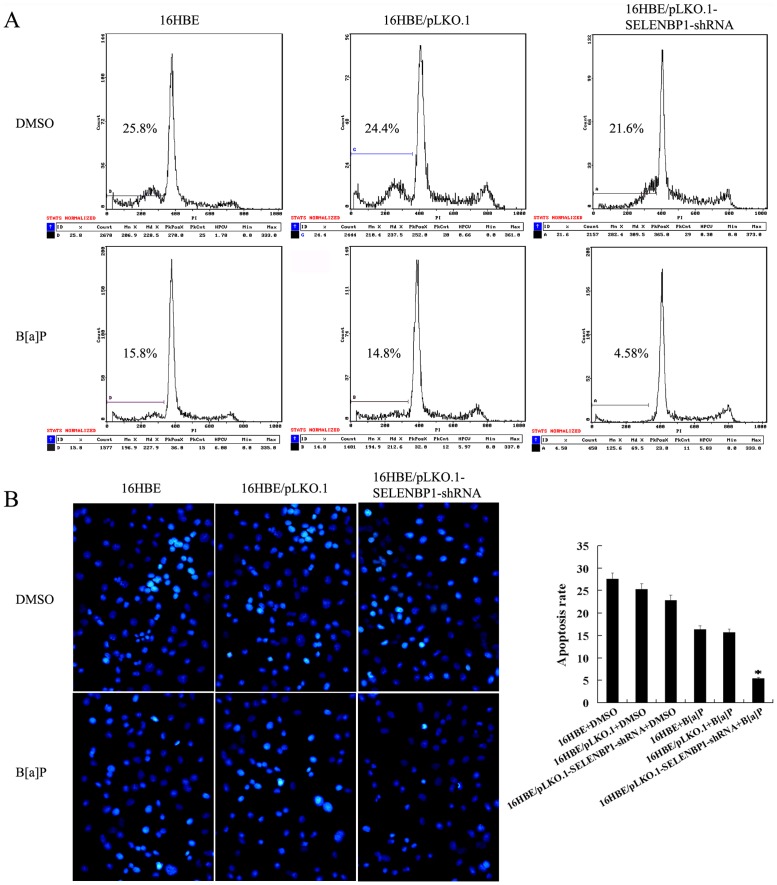
The effects of SELENBP1 knockdown on the apoptosis of B[a]P-transformed human bronchial epithelial cells. A, a representative result of flow cytometry analysis of cell apoptosis cultured in serum free medium after exposed to B[a]P for 18 weeks. Cells were grown in serum free DMEM medium for 24 h, and then assessed for apoptosis by flow cytometry as described in “Materials and Methods”. B, (*right*) Hoechst 33258 staining of cell apoptosis cultured in serum free medium after exposed to B[a]P for 18 weeks. Cells were grown in serum free DMEM medium for 24 h, and then assessed for apoptosis using the cell-permeable DNA dye Hoechst 33258. Apoptotic nuclei showing intense fluorescence corresponding to chromatin condensation; (*left*) a histogram showed the cell apoptotic rates. Three experiments were done; columns, mean; bars, S.D.(*, *P*<0.05 *vs.* untransfected or empty vector -transfected 16HBE cells exposed to B[a]P). Apoptosis of the cells exposed to vehicle (DMSO) for 18 weeks is also shown and used as controls. 16HBE, untransfected cells; 16HBE/pLKO.1, empty vector pLKO.1-transfected cells; 16HBE/pLKO.1-SELENBP1-shRNA, pLKO.1-SELENBP1-shRNA-tansfected cells.

To confirm that downregulation of SELENBP1 is involved in bronchial epithelial carcinogenesis, we generated stably transfected human bronchial epithelial cell line 16HBE cells with knockdown of SELENBP1 (Figure 3A), and measured the susceptibility of the transfected 16HBE cell transformation induced by B[a]P. After repeated treatment with 1 µm B[a]P for 18 weeks, significant differences in transformation efficiency between 16HBE cells with knockdown of SELENBP1 and control cells (empty vector-transfected 16HBE cells and untransfected cells) were seen: (1) MTT assay showed that cell growth rate in low serum medium is significantly higher in 16HBE cells with knockdown of SELENBP1 than in control cells (Figure 3B); (2) anchorage dependent and independent colony formation assays showed that about 1.6-fold more plate colonies and about 2.3-fold more soft agar colonies developed from 16HBE cells with knockdown of SELENBP1 compared with control cells(Figure 3C and D); (3) flow cytometric analysis revealed that a significant increase of S phase populations with a corresponding decrease of G0/G1 phase in 16HBE cells with knockdown of SELENBP1 compared with the control cells (Table 3); and (4) both Hoechst 33258 staining and flow cytometric analysis of apoptotic cells showed significantly less apoptotic cells are detected in 16HBE cells with knockdown of SELENBP1 than in the control cells (Figure 4). Taken together, these results demonstrated that knockdown of SELENBP1 increased the susceptibility of human bronchial epithelial cell transformation induced by B[a]P, supporting that SELENBP1 downregulation is involved in the carcinogenesis of human bronchial epithelial cells.

**Table 3:** Cell cycle distribution of 16HBE with knockdown of SELENBP1 and controls after repeated treatment with 1 µm B[a]P or DMSO for 18 weeks.

Cell lines	Number of cell (%)		
	G_0_/G_1_	S	G_2_/M
16HBE+DMSO	70.02±4.67	18.58±3.48	11.39±1.63
16HBE/pLKO.1+DMSO	69.10±3.51	19.64±2.87	11.25±1.65
16HBE/pLKO.1-SELENBP1-shRNA+ DMSO	65.79±3.59	21.96±2.58	12.25±2.22
16HBE+ B[a]P	67.94±2.15	19.10±2.44	12.96±2.13
16HBE/pLKO.1+ B[a]P	67.25±4.20	19.64±2.58	13.11±1.81
16HBE/pLKO.1− SELENBP1-shRNA+ B[a]P	50.56±5.24*	35.75±4.49*	13.69±1.55

* *P*<0.05 by T*-*test, 16HBE/pLKO.1-SELENBP1-shRNA+B[a]P *vs.* 16HBE/pLKO.1+B[a]P, 16HBE+ B[a]P, and 16HBE/pLKO.1-SELENBP1-shRNA+B[a]P *vs.* 16HBE +DMSO.

## Discussion

LSCC carcinogenesis is a multistage process from normal to preneoplastic lesions and then on to carcinoma [4]. Identification of proteins with altered expression in human bronchial epithelial carcinogenesis is important for understanding LSCC carcinogenic mechanisms, and discovering biomarkers for early detection of LSCC. In our study, iTRAQ labeling combined with 2D LC-MS/MS was used to identify differentially expressed proteins during human bronchial epithelial carcinogenesis, finding that SELENBP1 was progressively decreased during this process.

To validate the expressional change of SELENBP1 during human LSCC carcinogenic process found by proteomics, Western blotting and immunohistochemical analysis were performed to detect SELENBP1 expression in independent sets of tissue samples. The results showed that SELENBP1 expression was progressively decreased along with evolution of bronchial epithelial carcinogenesis, which is consistent with the findings in proteomic analysis, suggesting that the downregulation of SELENBP1 not only is involved in human bronchial epithelial carcinogenesis, but also is an early event in LSCC tumorigenesis. To our knowledge, this is the first time show that decreased SELENBP1 is an early event in LSCC carcinogenesis. Next, we evaluated the ability of SELENBP1 protein in distinguish NBE from preneoplastic lesions from invasive LSCC, finding that the expressional levels of SELENBP1 protein discriminate NBE from preneoplastic lesions from invasive LSCC with high sensitivity and specificity, suggesting that SELENBP1 is a potential biomarker for early detection of LSCC.

Selenium is a trace element that is essential for a number of biological processes. A deficiency of dietary selenium is associated with an increased incidence of epithelial cancers including lung, liver, colorectal, and prostate cancer [10]. Supplementation of dietary selenium for cancer prevention resulted in significant reduction in incidence of lung and other epithelial cancers [11]. SELENBP1, a member of selenoproteins family, has been shown to bind selenium covalently [7], [8], and mediate the intracellular transport of selenium [9]. Selenium exerts its anticarcinogenic effects mainly through selenoproteins at nutritional levels. SELENBP1 is expressed in various types of tissues, and its expression is reduced markedly in multiple epithelial cancers compared with their corresponding normal tissues, suggesting a possible link to malignancies associated with selenium deficiencies [25]–[28]. Furthermore, reduced SELENBP1 was also associated with poor outcome and differentiation in lung [25], colorectal [26], ovarian [27], and prostate [28] cancer. Therefore, reduced SELENBP1 may play a critical role in regulating malignant transformation and cancer progression. However, there is little information on the expression and function of SELENBP1 during human LSCC carcinogenic process.

Polycyclic aromatic hydrocarbons such as B[a]P are main lung carcinogens within tobacco smoke [12], and the source of DNA adducts [13]. It is known that selenium can inhibit carcinogen-induced covalent DNA adduct formation; impede oxidative damage to DNA; increase cell apoptosis, accelerate cell senescence;and inhibit cell proliferation [14]–[17], indicating selenium can antagonize B[a]P-induced tumorigenesis. It has been reported that selenium activated the DNA repair branch of the p53 pathway, induced nucleotide excision DNA repair, and protected normal human or mouse fibroblasts from UV-radiation [18], [19]. Recent advances showed that selenium can activate early barriers of tumorigenesis, namely senescence and DNA damage response, by rapidly activating ATM, which in turn initiates a cascade of DNA damage response and cellular senescence in non-cancerous and cancer cells [20], [21]. These studies further support that selenium can antagonize B[a]P-induced tumorigenesis.

As the anticarcinogenic effects of selenium possibly are mediated by SELENBP1, we further explore SELENBP1 downregulation is involved in LSCC via increasing the susceptibility of human bronchial epithelial cells to B[a]P-induced tumorigenesis. We knocked down SELENBP1 in immortalized human bronchial epithelial line 16HBE cells, and then detected whether SELENBP1 knockdown increased the susceptibility of cell transformation induced by carcinogen B[a]P. After weekly exposed to 1 µm B[a]P for 18 weeks, transformation efficiency of 16HBE cells with SELENBP1 knockdown was significantly higher than that of control cells, and SELENBP1 knockdown increased the susceptibility of bronchial epithelial cell transformation induced by B[a]P. The results not only demonstrate that decreased SELENBP1 plays an important role in human bronchial epithelial carcinogenesis, but also support that SELENBP1 might mediate anti-LSCC effects of selenium. To our knowledge, this is the first time show that decreased SELENBP1 is related to benzo(a)pyrene-induced LSCC tumorigenesis.

As downregulation of SELENBP1 is an early event in LSCC, and renders the human bronchial epithelial cells susceptible to B[a]P-induced carcinogenesis, we suggest that the detection of SELENBP1 expression can be used for monitoring high risk group of LSCC including smokers, and improving the early diagnosis of LSCC, and supplementation of dietary selenium may be significant for the prevention of LSCC.

In conclusion, we identified and confirmed the progressive decrease of SELENBP1 in the human bronchial epithelial carcinogenic process, found that the expressional levels of SELENBP1 can discriminate normal bronchial epithelium from preneoplastic lesions from invasive LSCC. We further showed that SELENBP1 knockdown increased the susceptibility of bronchial epithelial cell transformation induced by B[a]P. The findings reported here could have clinical value in monitoring high risk group of LSCC including smokers, and improving the early diagnosis of LSCC.

## Supporting Information

Table S1 **Characteristics of formalin-fixed and paraffin-embedded archival tissue specimens.**
(DOC)

Table S2 **Differentially expressed proteins during bronchial epithelial carcinogenesis.**
(DOC)
