# Supplementary material for: The Function and Significance of SELENBP1 Downregulation in Human Bronchial Epithelial Carcinogenic Process
Source: PLoS One. 2013 Aug 19;8(8):e71865. doi: 10.1371/journal.pone.0071865 (PMC3747066; doi:10.1371/journal.pone.0071865)
Supplement: Table S1 — Characteristics of formalin-fixed and paraffin-embedded archival tissue specimens. (DOC) [file pone.0071865.s001.doc]

**Table S1 Characteristics of formalin-fixed and paraffin-embedded archival tissue specimens**

| Patients | NBE (66) | | SM (64) | AH (60) | CIS (13) | LSCC(66) |
| --- | --- | --- | --- | --- | --- | --- |
| Age | | 54±8 | 53±9 | 56±10 | 55±9 | 58±10 |
| Sex | |  |  |  |  |  |
| Male | | 31 | 34 | 33 | 7 | 36 |
| Female | | 35 | 30 | 27 | 6 | 30 |
| Smoking status | |  |  |  |  |  |
| Nonsmoker | | 35 | 31 | 29 | 6 | 32 |
| Ex-smoker | | 5 | 6 | 7 | 2 | 9 |
| smoker | | 26 | 27 | 24 | 5 | 25 |
| With LSCC | | 10 | 20 | 36 | 9 | 66 |
| Without LSCC | | 56 | 44 | 24 | 4 | 0 |

LSCC, lung squamous cell carcinoma
