# Supplementary material for: The Function and Significance of SELENBP1 Downregulation in Human Bronchial Epithelial Carcinogenic Process
Source: PLoS One. 2013 Aug 19;8(8):e71865. doi: 10.1371/journal.pone.0071865 (PMC3747066; doi:10.1371/journal.pone.0071865)
Supplement: Table S2 — Differentially expressed proteins during bronchial epithelial carcinogenesis. (DOC) [file pone.0071865.s002.doc]

**Table S2 Differentially expressed proteins during bronchial epithelial carcinogenesis**

| **No** | **Accession #** | **Protein Name** | **SM vs.NBE** | **AH/CIS vs.SM** | **LSCC vs.AH/CIS** | **LSCC vs.NBE** |
| --- | --- | --- | --- | --- | --- | --- |
| 1 | IPI00029733.1 | Aldo-keto reductase family 1 member C1 |  | ↑1.600 | ↑58.824 | ↑35.714 |
| 2 | IPI00025512.2 | Heat shock protein beta-1 | ↑3.636 | ↑1.742 | ↑4.878 | ↑20.000 |
| 3 | IPI00221222.7 | Activated RNA polymerase II transcriptional coactivator p15 | ↑3.623 |  | ↑5.076 | ↑20.000 |
| 4 | IPI00027462.1 | Protein S100-A9 | ↑6.757 | ↑2.632 | ↑1.563 | ↑15.625 |
| 5 | IPI00411765.3 | Isoform 2 of 14-3-3 protein sigma | ↑12.821 | ↑1.645 | ↓0.484 | ↑9.434 |
| 6 | IPI00010214.1 | Protein S100-A14 | ↑3.663 |  | ↑1.996 | ↑7.937 |
| 7 | IPI00217963.3 | type I cytoskeletal 16 | ↓0.473 | ↑27.027 | ↓0.395 | ↑7.143 |
| 8 | IPI00375746.4 | Isoform 1 of Guanylate-binding protein 6 | ↑1.859 | ↑1.610 | ↑1.901 | ↑5.525 |
| 9 | IPI00017334.1 | Prohibitin |  |  | ↑4.167 | ↑5.319 |
| 10 | IPI00453473.6 | Histone H4 | ↑2.695 | ↑8.475 |  | ↑4.854 |
| 11 | IPI00218988.4 | Isoform 2 of Adenylate kinase isoenzyme 2, mitochondrial | ↑2.488 |  | ↑4.219 | ↑4.545 |
| 12 | IPI00738499.2 | Ferritin light chain |  |  | ↑3.534 | ↑4.444 |
| 13 | IPI00032140.4 | Serpin H1 precursor |  |  | ↑3.356 | ↑4.329 |
| 14 | IPI00549725.6 | Phosphoglycerate mutase 1 |  | ↓0.418 | ↑5.848 | ↑4.184 |
| 15 | IPI00013933.2 | Isoform DPI of Desmoplakin | ↑3.049 | ↑2.151 |  | ↑3.717 |
| 16 | IPI00887678.1 | LOC654188 similar to peptidylprolyl isomerase A-like | ↑2.304 |  | ↑1.754 | ↑3.704 |
| 17 | IPI00021926.2 | 26S protease regulatory subunit S10B | ↑2.242 |  | ↑3.106 | ↑3.559 |
| 18 | IPI00018873.1 | Isoform 1 of Nicotinamide phosphoribosyltransferase |  | ↑5.882 |  | ↑3.509 |
| 19 | IPI00021347.1 | Ubiquitin-conjugating enzyme E2 L3 | ↓0.509 | ↑5.208 | ↑3.195 | ↑3.448 |
| 20 | IPI00300725.7 | type II cytoskeletal 6A | ↑31.250 | ↑3.731 | ↓0.052 | ↑3.413 |
| 21 | IPI00024933.3 | 60S ribosomal protein L12 |  |  | ↑2.208 | ↑3.356 |
| 22 | IPI00219018.7 | Glyceraldehyde-3-phosphate dehydrogenase | ↑2.695 |  |  | ↑3.257 |
| 23 | IPI00009866.6 | type I cytoskeletal 13 | ↑30.303 | ↓0.622 |  | ↑3.185 |
| 24 | IPI00642971.3 | eukaryotic translation elongation factor 1 delta isoform 1 |  |  | ↑1.927 | ↑2.857 |
| 25 | IPI00007047.1 | Protein S100-A8 | ↑2.079 | ↑1.969 |  | ↑2.857 |
| 26 | IPI00646656.2 | Asparaginyl-tRNA synthetase variant (Fragment) |  |  | ↑3.030 | ↑2.786 |
| 27 | IPI00644989.2 | Isoform 1 of Protein disulfide-isomerase A6 precursor |  |  | ↑2.618 | ↑2.747 |
| 28 | IPI00015842.1 | Reticulocalbin-1 precursor |  |  | ↑2.725 | ↑2.674 |
| 29 | IPI00789551.1 | Uncharacterized protein MATR3 |  |  | ↑3.788 | ↑2.545 |
| 30 | IPI00294834.6 | Aspartyl/asparaginyl beta-hydroxylase |  |  |  | ↑2.427 |
| 31 | IPI00746438.2 | Isoform 2 of 60S ribosomal protein L11 |  |  |  | ↑2.183 |
| 32 | IPI00295400.1 | Tryptophanyl-tRNA synthetase | ↓0.414 |  | ↑4.405 | ↑2.179 |
| 33 | IPI00003362.2 | HSPA5 protein |  |  | ↑3.003 | ↑2.174 |
| 34 | IPI00026230.1 | Heterogeneous nuclear ribonucleoprotein H2 | ↑1.812 |  | ↑1.748 | ↑2.146 |
| 35 | IPI00465315.6 | Cytochrome c |  |  | ↑1.672 | ↑2.079 |
| 36 | IPI00011654.2 | Tubulin beta chain | ↑1.838 |  |  | ↑2.037 |
| 37 | IPI00747533.2 | PGD 56 kDa protein |  |  |  | ↑1.946 |
| 38 | IPI00024284.4 | Basement membrane-specific heparan sulfate proteoglycan core protein precursor |  | ↑1.506 | ↑2.146 | ↑1.931 |
| 39 | IPI00748905.1 | NAPB protein |  |  |  | ↑1.894 |
| 40 | IPI00796333.1 | ALDOA 45 kDa protein |  |  | ↑2.445 | ↑1.873 |
| 41 | IPI00027497.5 | Glucose-6-phosphate isomerase | ↓0.628 |  |  | ↑1.818 |
| 42 | IPI00291006.1 | Malate dehydrogenase | ↑1.592 |  |  | ↑1.815 |
| 43 | IPI00848342.1 | Lactotransferrin precursor |  |  |  | ↑1.789 |
| 44 | IPI00658109.1 | LOC100133623 Creatine kinase |  |  |  | ↑1.709 |
| 45 | IPI00003519.1 | 116 kDa U5 small nuclear ribonucleoprotein component |  |  | ↑1.786 | ↑1.701 |
| 46 | IPI00218319.3 | Isoform 2 of Tropomyosin alpha-3 chain |  |  |  | ↑1.570 |
| 47 | IPI00296534.1 | Isoform D of Fibulin-1 precursor | ↓0.641 |  | ↑1.898 | ↑1.517 |
| 48 | IPI00009032.1 | Lupus La protein |  |  |  | ↓0.655 |
| 49 | IPI00375145.1 | Isoform Short of Ubiquitin carboxyl-terminal hydrolase 5 |  |  | ↓0.473 | ↓0.629 |
| 50 | IPI00450768.7 | type I cytoskeletal 17 | ↑3.247 | ↑8.333 | ↓0.056 | ↓0.603 |
| 51 | IPI00220766.5 | Lactoylglutathione lyase |  |  | ↓0.595 | ↓0.566 |
| 52 | IPI00014898.2 | Isoform 1 of Plectin-1 |  |  | ↓0.430 | ↓0.498 |
| 53 | IPI00472724.1 | Elongation factor 1-alpha-like 3 |  |  | ↓0.256 | ↓0.462 |
| 54 | IPI00020599.1 | Calreticulin precursor |  |  |  | ↓0.452 |
| 55 | IPI00303476.1 | ATP synthase subunit beta, mitochondrial precursor |  |  | ↓0.476 | ↓0.440 |
| 56 | IPI00027107.5 | Tu translation elongation factor, mitochondrial precursor |  |  |  | ↓0.432 |
| 57 | IPI00411704.9 | Isoform 1 of Eukaryotic translation initiation factor 5A-1 |  |  |  | ↓0.403 |
| 58 | IPI00440493.2 | ATP synthase subunit alpha |  |  | ↓0.496 | ↓0.403 |
| 59 | IPI00853547.1 | glucose-6-phosphate dehydrogenase isoform a |  | ↑2.058 | ↓0.141 | ↓0.368 |
| 60 | IPI00183695.9 | Protein S100-A10 | ↑2.049 | ↑2.326 | ↓0.187 | ↓0.352 |
| 61 | IPI00027444.1 | Leukocyte elastase inhibitor | ↑1.730 |  | ↓0.603 | ↓0.348 |
| 62 | IPI00465084.6 | Desmin |  |  | ↓0.335 | ↓0.340 |
| 63 | IPI00017704.3 | Coactosin-like protein |  |  | ↓0.327 | ↓0.333 |
| 64 | IPI00465431.7 | Galectin-3 |  | ↓0.620 | ↓0.601 | ↓0.323 |
| 65 | IPI00218918.5 | Annexin A1 |  |  | ↓0.212 | ↓0.320 |
| 66 | IPI00003865.1 | Isoform 1 of Heat shock cognate 71 kDa protein |  |  |  | ↓0.302 |
| 67 | IPI00022793.5 | Trifunctional enzyme subunit beta |  |  |  | ↓0.289 |
| 68 | IPI00291136.4 | Collagen alpha-1(VI) chain precursor |  |  |  | ↓0.283 |
| 69 | IPI00010471.5 | Plastin-2 |  |  | ↓0.367 | ↓0.263 |
| 70 | IPI00216691.5 | Profilin-1 |  |  |  | ↓0.250 |
| 71 | IPI00644087.1 | LMNA Progerin |  |  | ↓0.212 | ↓0.240 |
| 72 | IPI00018219.1 | Transforming growth factor-beta-induced protein ig-h3 precursor | ↑1.855 |  |  | ↓0.234 |
| 73 | IPI00180675.4 | Tubulin alpha-1A chain | ↓0.448 |  |  | ↓0.224 |
| **74** | **IPI00745729.2** | **SELENBP1 54 kDa protein** | **↓0.643** | **↓0.565** | **↓0.525** | **↓0.223** |
| 75 | IPI00792011.1 | Calcyphosin | ↓0.223 |  |  | ↓0.212 |
| 76 | IPI00022200.2 | alpha 3 type VI collagen isoform 1 precursor |  | ↓0.510 |  | ↓0.203 |
| 77 | IPI00329801.12 | Annexin A5 | ↓0.423 | ↑1.565 | ↓0.261 | ↓0.189 |
| 78 | IPI00455383.4 | Isoform 2 of Clathrin heavy chain 1 |  |  | ↓0.191 | ↓0.177 |
| 79 | IPI00000105.4 | Major vault protein |  | ↓0.627 | ↓0.214 | ↓0.168 |
| 80 | IPI00414320.1 | Annexin A11 |  | ↓0.437 |  | ↓0.166 |
| 81 | IPI00291005.8 | Malate dehydrogenase |  |  |  | ↓0.164 |
| 82 | IPI00022204.2 | Isoform 1 of Serpin B3 |  | ↓0.337 |  | ↓0.130 |
| 83 | IPI00219757.13 | Glutathione S-transferase P | ↓0.408 | ↓0.512 | ↓0.507 | ↓0.124 |
| 84 | IPI00654709.1 | ALDH3A1 protein (Fragment) |  |  |  | ↓0.122 |
| 85 | IPI00745872.2 | Isoform 1 of Serum albumin precursor |  | ↓0.493 | ↓0.250 | ↓0.120 |
| 86 | IPI00872684.1 | EZR 69 kDa protein | ↓0.548 |  | ↓0.217 | ↓0.109 |
| 87 | IPI00009123.1 | Nucleobindin-2 precursor |  | ↓0.235 |  | ↓0.102 |
| 88 | IPI00027463.1 | Protein S100-A6 | ↓0.639 |  | ↓0.224 | ↓0.101 |
| 89 | IPI00887291.1 | hypothetical LOC729659 |  |  | ↓0.071 | ↓0.095 |
| 90 | IPI00553177.1 | Isoform 1 of Alpha-1-antitrypsin precursor |  |  | ↓0.065 | ↓0.078 |
| 91 | IPI00152295.1 | Spermatogenesis-associated protein 18 | ↓0.276 |  | ↓0.554 | ↓0.077 |
| 92 | IPI00847342.1 | keratin 7 |  |  | ↓0.088 | ↓0.073 |
| 93 | IPI00024915.2 | Isoform Mitochondrial of Peroxiredoxin-5 | ↓0.470 | ↓0.246 |  | ↓0.072 |
| 94 | IPI00007427.2 | AGR2 |  | ↓0.223 | ↓0.165 | ↓0.071 |
| 95 | IPI00554788.5 | type I cytoskeletal 18 |  |  | ↓0.096 | ↓0.071 |
| 96 | IPI00000816.1 | 14-3-3 protein epsilon |  |  | ↓0.076 | ↓0.060 |
| 97 | IPI00294739.1 | SAM domain and HD domain-containing protein 1 | ↓0.642 |  |  | ↓0.051 |
| 98 | IPI00554648.3 | type II cytoskeletal 8 |  | ↓0.536 | ↓0.066 | ↓0.047 |
| 99 | IPI00807545.1 | Isoform 3 of Heterogeneous nuclear ribonucleoprotein K |  |  | ↓0.044 | ↓0.046 |
| 100 | IPI00022977.1 | Creatine kinase B-type | ↓0.388 | ↓0.422 | ↓0.171 | ↓0.045 |
| 101 | IPI00795633.1 | CLU |  |  | ↓0.047 | ↓0.044 |
| 102 | IPI00640817.1 | AK1 Adenylate kinase 1 |  |  | ↓0.072 | ↓0.040 |
